# HIV-1 subtype diversity and immuno-virological outcomes among adolescents failing antiretroviral therapy in Cameroon: A cohort study

**DOI:** 10.1371/journal.pone.0293326

**Published:** 2023-10-25

**Authors:** Willy Le roi Togna Pabo, Joseph Fokam, Debimeh Njume, Désiré Takou, Maria-Mercedes Santoro, Raymond Babila Nyasa, Collins Chenwi, Marie Laure Mpouel, Grace Beloumou, Ezechiel Semengue Ngoufack Jagni, Alex Durand Nka, Aude Christelle Ka’e, Georges Teto, Beatrice Dambaya, Sandrine Djupsa, Davy Hyacinthe Gouissi Anguechia, Molimbou Evariste, Cedric Kamta, Lionel Bala, Virginie Lambo, Edie Gregory Halle-Ekane, Vittorio Colizzi, Carlo Federico Perno, Alexis Ndjolo, Roland Ndip Ndip

**Affiliations:** 1 Chantal BIYA International Reference Centre for Research on HIV/AIDS prevention and management, Yaoundé, Cameroon; 2 Faculty of Medicine and Biomedical Sciences, Yaoundé, Cameroon; 3 Faculty of Science, University of Buea, Buea, Cameroon; 4 Faculty of Health Sciences, University of Buea, Buea, Cameroon; 5 National HIV Drug Resistance Group, Ministry of Public Health, Yaoundé, Cameroon; 6 University of Rome Tor Vergata, Rome, Italy; 7 Evangelic University of Cameroon, Bandjoun, Cameroon; 8 Mfou District Hospital, Mfou, Cameroon; 9 Mbalmayo District Hospital, Mbalmayo, Cameroon; 10 Nkomo Integrated Health Center, Nkomo, Cameroon; 11 Bambino Gesu Pediatric Hospital, Rome, Italy; University of Cincinnati College of Medicine, UNITED STATES

## Abstract

**Objective:**

We sought to evaluate the variability of HIV-1 and its effect on immuno-virological response among adolescents living with perinatally acquired HIV (APHI).

**Methods:**

A cohort study was conducted from 2018–2020 among 311 APHI receiving antiretroviral therapy (ART) in Cameroon. Sequencing of protease and reverse transcriptase regions was performed for participants experiencing virological failure, VF, (Plasma viral load, PVL ≥ 1000 RNA copies/ml). HIV-1 subtypes were inferred by phylogeny; immuno-virological responses were monitored at 3-time points (T1-T3). Cox regression modeling was used to estimate adjusted hazard ratios (aHRs) of progression to: CD4 < 250, and PVL > 5log_10_, adjusted for acquired drug resistance, gender, ART line, adherence, and duration on treatment; p < 0.05 was considered statistically significant.

**Results:**

Of the 141 participants in VF enrolled, the male-female ratio was 1:1; mean age was 15 (±3) years; and median [IQR] duration on ART was 51 [46–60] months. In all phases, 17 viral clades were found with a predominant CRF02_AG (58.2%, 59.4%, and 58.3%). From T1-T3 respectively, there was an increasing CD4 count (213 [154–313], 366 [309–469], and 438 [364–569] cells/mm^3^) and decline log_10_ PVL (5.23, 4.43, and 4.43), similar across subtypes. Among participants with CRF02_AG infection, duration of treatment was significantly associated with both rates of progression to CD4 < 250, and PVL > 5log_10_, aHR = 0.02 (0.001–0.52), and aHR = 0.05 (0.01–0.47) respectively. Moreover, four potential new HIV-1 recombinants were identified (CRF02_AG/02D, CRF02_AG/02A1F2, D/CRF02_AG, and AF2/CRF02_AG), indicating a wide viral diversity.

**Conclusion:**

Among APHI in settings like Cameroon, there is a wide genetic diversity of HIV-1, driven by CRF02_AG and with potential novel clades due to ongoing recombination events. Duration of treatment significantly reduces the risk of disease progression.

## 1. Introduction

HIV molecular surveillance reveals recombinant forms account for almost a quarter of HIV infection worldwide [1]. West-Central Africa is believed to be the geographical origin of HIV-1, with the Congo basin being the most likely birthplace of HIV-1 groups M, N, O, and P [2, 3]. This is perhaps the reason why it has one of the most genetically diverse HIV epidemics on the globe. Moreover, the circulation of virtually every known HIV-1 group M pure subtype, many circulating recombinant forms (CRFs), and a variety of unique recombinant forms (URFs) has been observed within this geographical region [4]. The most widely circulating variant in this region is the CRF02_AG viral clade.

There is increasing evidence that the extreme variability and high evolution rate of HIV-1 favors the emergence of HIV antiretroviral drug resistance (HIVDR). In addition, some recombinant forms, such as the CRF01_AE variant in China, have been shown to have a faster rate of disease progression, and better replicative fitness when compared to pure lineages [5]. In a context of wide genetic diversity among adolescents living with perinatally acquired HIV (APHI) [6], it is therefore essential to monitor and genetically characterize HIV on a global scale. Special attention has to be paid to hot spot regions with wide HIV genetic variability like Cameroon, noting that subtype variability and the extent of viral diversity within a geographical region considerably impact HIV diagnosis, treatment outcome, and hence prevention and management [4, 7, 8].

The increase in HIV-1 viral diversity because of the relative adaptation of the virus to a given host environment constitutes a factor that is particularly relevant to the emergence of drug-resistant variants. This provides a unique opportunity to study and monitor the evolution of HIV-1 strains and the clinical implications of these on the treatment, and immune/virological response among adolescents [9–11].

The determination of HIV subtypes is based on comparing a query sequence to a set of reference sequences and obtaining the best match as the putative subtype. A wide array of currently used subtyping tools rely on an initial alignment step to measure similarity with the reference set. The population of APHI is of particular interest because of the long time after infection (starting from an established time point, that is the birth), and as such viral adaptation to the host is likely to occur.

In this study, we evaluated the effect of HIV-1 genetic diversity on immune-virological response and described potential emerging resistant variants among APHI failing first- and second-line antiretroviral treatment. Specifically, we sought to assess HIV-1 genetic variability among these APHI, evaluate the immune-virological response according to HIV-1 subtypes, determine the trends of virological response according to HIV-1 subtypes, and describe the selection of potential resistant variants among these adolescents.

## 2. Materials and methods

### 2.1 Study design

A cohort study was conducted from 2018–2020 among 311 adolescents living with perinatally acquired HIV (APHI) receiving antiretroviral therapy (ART) in one of the selected health facilities within the *“Resistance Evolution among Adolescents in Yaoundé and its surroundings” (READY-study)* in the Centre region of Cameroon. Participants were recruited following consecutive, and exhaustive sampling. Follow-up was performed at enrollment (T1), at 6 months (T2), and at 12 months (T3). Included in the current analysis were only participants failing first- and second-line ART with plasma viral load (PVL) ≥ 1000 RNA copies/ml (Table 1).

**Table 1:** Biological and clinical characteristics of the EDCTP-Ready study parent cohort.

	Enrolment (T1)		6-months (T2)		12-months (T3)	
	Frequency	Percentage	Frequency	Percentage	Frequency	Percentage
	n = 311	(%)	n = 272	(%)	n = 243	(%)
**Age (years)**						
10–14	169	54.3	108	39.7	102	42.3
15–19	142	45.7	164	60.3	139	57.7
**Gender**						
Male	142	46.1	124	45.6	105	43.8
Female	166	53.9	148	54.4	135	56.2
**Clinical stage**						
I/II	286	94.7	245	90.1	199	90.9
III/IV	16	5.3	27	9.9	20	9.1
**ART line**						
First	256	83.9	181	68.8	129	58.1
Second	49	16.1	82	31.2	93	41.9
**Adherence**						
Good	196	66.4	158	58.3	153	66.5
Poor	99	33.6	113	41.7	77	33.5
**CD4 classes**						
≥250	202	74.5	215	81.1	211	90.2
<250	69	25.5	50	18.9	23	9.8
**PVL classes**						
≥1000	121	39.7	105	39.9	68	28.2
<1000	184	60.3	158	60.1	173	71.8
**Sequencing success rate**	55/121	45.5	96/105	91.4	60/68	88.2
**ART regimen**						
**First line RTI-/INSTI-based** *						
ABC + 3TC + EFV	54	17.4	41	15.1	17	7.0
ABC + 3TC + NVP	1	0.3	-	-	-	-
AZT + 3TC + DTG	-	-	-	-	1	0.4
AZT + 3TC + EFV	31	10.0	16	5.9	11	4.5
AZT + 3TC + NVP	78	25.1	53	19.5	37	15.2
D4T + 3TC + NVP	4	1.3	-	-	-	-
TDF + 3TC + EFV	85	27.3	71	26.1	68	28.0
TDF + 3TC + NVP	3	1.0	1	0.4	2	0.8
**Second line PI/r-based** **						
ABC + 3TC + ATV/r	5	1.6	6	2.2	6	2.5
ABC + 3TC + LPV/r	6	1.9	7	2.6	6	2.5
ABC + AZT + LPV/r	1	0.3	-	-	-	-
ABC + DDI + LPV/r	1	0.3	-	-	-	-
AZT + 3TC + ATV/r	2	0.6	3	1.1	8	3.3
AZT + 3TC + LPV/r	2	0.6	10	3.7	13	5.3
AZT + DDI + LPV/r	1	0.3	-	-	-	-
TDF + 3TC+ ATV/r	19	6.1	43	15.8	57	23.5
TDF + 3TC + ATV/r + DTG	-	-	-	-	1	0.4
TDF + 3TC + LPV/r	10	3.2	12	4.4	10	4.1
TDF + ABC + LPV/r	1	0.3	-	-	-	-
TDF + DDI + LPV/r	1	0.3	-	-	-	-

*Reverse transcriptase inhibitor-/ integrase strand transfer inhibitor-based antiretroviral therapy.

**Ritonavir boosted protease inhibitor-based second line antiretroviral therapy.

### 2.2 Inclusion and exclusion criteria

Included in the study were APHI; aged 10–19 years, receiving a standard reverse transcriptase inhibitor-based first- or ritonavir-boosted protease inhibitor-based second-line ART regimen for at least 6 months, provided written assent, and informed consent from their legal guardian(s), and irrespective of exposure to the prevention of mother to child transmission (PMTCT) antiretrovirals. APHI who were not formally registered in the ART monitoring system of any study site, reported to be ART-naïve, or on a drug regimen not included in the national guidelines, or structured treatment interruption were not enrolled. Moreover, participants who freely withdrew from the study, or were transferred out of a study site before mid- or endpoint were excluded.

### 2.3 Sample size

The minimal sample size was established following statistical calculations [12]. Assuming the rate of VF at 40%, a 95% confidence interval, and 80% statistical power, the sample size was 174 participants. Adding 10% potential loss to follow-up (LTFU) during the one-year study period and 20% sequencing failure rate, the minimal sample size was 243 APHI, majoring to 250 total sample size, further stratified into 150 APHI in the referral centers (75 per site), and 100 in the rural HIV management unit settings (50 per site), as per coverage in ART in these two geographical locations. Effectively 311 participants were enrolled, 213 from referral centers and 98 from management units. Of these, the current analysis included 141 participants (with PVL ≥ 1000 RNA copies/mL) who had sequence data available from enrolment to 12-month follow-up.

### 2.4 Laboratory procedure

Following enrolment, ten mL of whole blood were collected from each participant and transported on icepacks within 6 hours to the virology laboratory of the Chantal Biya international reference center (CIRCB), Yaoundé, Cameroon. From these, a whole blood aliquot (for CD4 enumeration) was gotten. Moreover, plasma aliquots (for PVL and HIV genotypic resistance testing, GRT) were obtained after centrifugation at 1600 rpm for 10 minutes and stored at −80°C for further analyses. CD4 cell count was performed using the Pima CD4 (Abbott/Pantech (Pty) Ltd, Westville, South Africa) automatic test as per the manufacturer’s instructions. PVL measurement was performed using the Abbott Applied Biosystem platform (Real-Time PCR AB m2000RT) as per the manufacturer’s instructions (Abbott Laboratories, USA), with a detection threshold of 40 copies/mL (lower) and 10,000,000 copies/mL (upper). To increase the sensitivity of the polymerase chain reaction (PCR) amplification, HIV-1 RNA extraction was performed from 1000 μL of plasma aliquots and an initial 2-hour refrigerated centrifugation step at 14 000 rpm to concentrate viral RNA was also performed. HIV-1 RNA was extracted manually from 140 μL of plasma using the QIAGEN protocol (QIAamp^®^ DNA Minikit; QIAGEN, Courtaboeuf, France). GRT was done when the viral load was > 1000 copies/mL using an in-house protocol [13]. Eight in-house primers (B, F, SEQ1, SEQ2, SEQ3, SEQ4, SEQ5, TAK3) that completely covered the protease and reverse-transcriptase regions in the pol gene fragment (~1600 base pairs) were used for sequencing. The latter were designed such that each zone of the fragment was covered by at least two primers. The primer sequences used were as follows:

“5'-AGC AGA CCA GAG CCA ACA GC-3 ” (2140–2159 gag);

“5'-CCA TCC AT T CCT GGC TTT AAT-3 '” (2582–2602 pol);

“5'-GAA GAA TGG ATG GCC CAA AA-3 '” (2590–2609 pol);

“5'-TTG TAC AGA AAA GGA AAA GGA AGG-3 '” (2660–2683 pol);

“5'-CCC TGT GGA AAG CAC ATT GTA-3 '” (2985–3004 pol, with one insertion);

“5'-GCT TCC ACA GGG ATG GAA-3 '” (2993–3011 pol);

“5'-CTA TTA AGT CTT ATG TTG GGT CA-3 '” (3506–3528 pol);

“5'-TT CCC CAT ATT ACT ATG CTT-3 '” (3683–3703 pol).

PCR products were sequenced using the Applied Biosystems 3500 genetic analyzer; the sequences thus obtained were edited using the Recall CDC Atlanta GA USA software and drug resistance mutations (DRMs) interpreted using Stanford HIVdb.v8.8; Subtyping was done using a rapid subtyping tool, Stanford HIVdb v8.9, and confirmed by MEGA X for molecular phylogeny using the Neighbor-Joining algorithm [14], maximum likelihood method [14] and a 500 replicates bootstrap value > 80% for subtype assignation. Assessment of potential emerging variants was performed using Splits Tree 4 v4.6 software [15] and genetic breakpoints were identified using the Recombinant Detection program version 4 (RDP4) [16]. Reference sequences included in the phylogenetic analysis were downloaded following a blast search from the National Center for Biotechnology Information website; https://www.ncbi.nlm.nih.gov/.

The major outcomes from the study were the viral genetic diversity among APHI, immune-virological responses stratified by subtype, the trends of virological response stratified by subtype, and the potential emerging resistant variants among these APHI failing treatment. Adequate immunological status was defined as absolute CD4 count ≥ 250 cells/mm^3^ and Immunological failure (IF) as < 250 CD4 cells/mm^3^ [17]. PVL was classified as high viral load (PVL > 5log_10_ RNA copies/ml), and low viral load (PVL < 5 log_10_) [18]. Genotypic resistance testing (GRT) was performed for these participants failing treatment.

### 2.5 Data analysis

Data were analyzed using SPSS version 22, with p < 0.05 considered statistically significant. A comparison of means and medians of various characteristics was done using the paired t-test. Chi-square and Fisher’s exact tests were used for determining associations between qualitative variables. Study endpoints were defined as CD4 count < 250 cells/mm^3^ and PVL > 5 log RNA/mL. Cox proportional hazard regression models were used to estimate hazard ratios (HRs) of disease progression to each endpoint stratified by HIV-1 subtype, adjusting for the presence of drug resistance mutations, gender, ART line, adherence, and duration on ART. Kaplan Meier curves were used to examine time to CD4 count decline to < 250 cells/mm^3^ and VL > 5 log_10_ RNA copies/mL on ART across subtypes, with the use of the log-rank test to test the significance of observed differences between groups. Survival curves were drawn using SPSS version 22. Moreover, median CD4 cell rise and PVL decline over time were determined and presented as figures.

### 2.6 Ethical statement

Administrative authorizations were obtained from the Directors of the health facilities used in this study. Moreover, an ethical clearance was obtained from the Cameroonian National ethics committee for research on human subjects (clearance № 2018/01/981/CE/CNERSH/SP) in Yaoundé, Cameroon. Written informed consent and assent were obtained from the parents or legal guardian(s), and every participant respectively. Laboratory results of viremia, CD4 cell count, and GRT were freely delivered to each participant for their clinical benefits, data management was under strict confidentiality by using unique identifiers, and access to data was password protected.

## 3. Results

**Fig 1:**
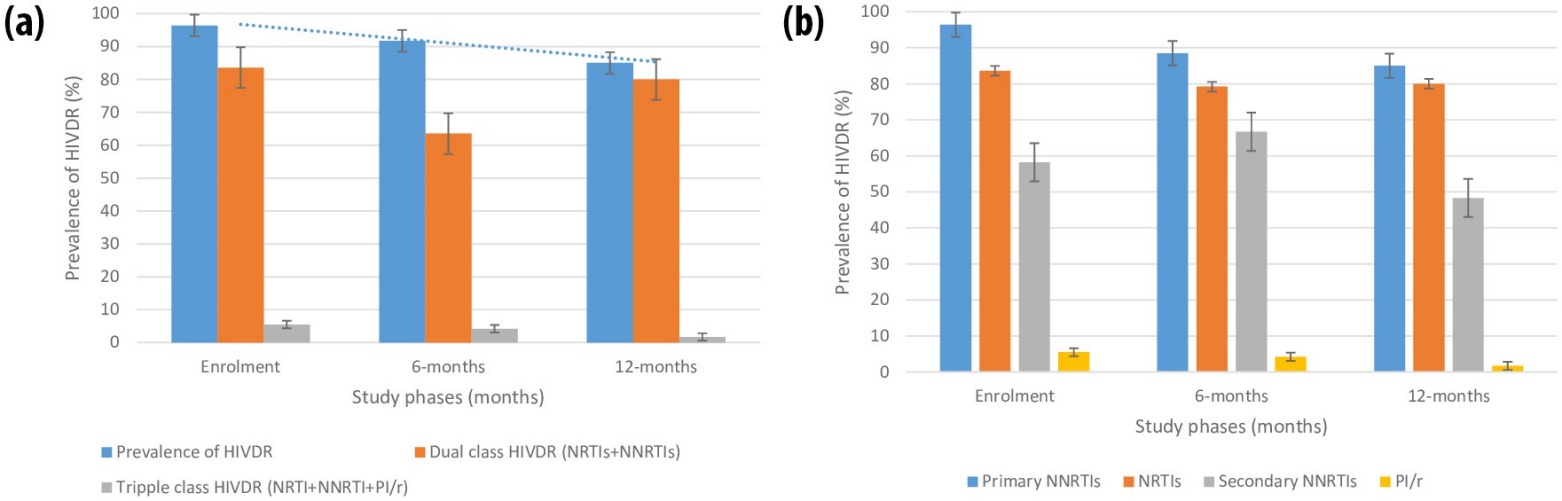
**a:** Trends of HIVDR across time points. Blue line: the decreasing trend of HIVDR over time. Error bars denote standard error.**b:** Trends of HIV drug resistance with respect to antiretroviral drug classes. Error bars denote standard error.

**Fig 2:**
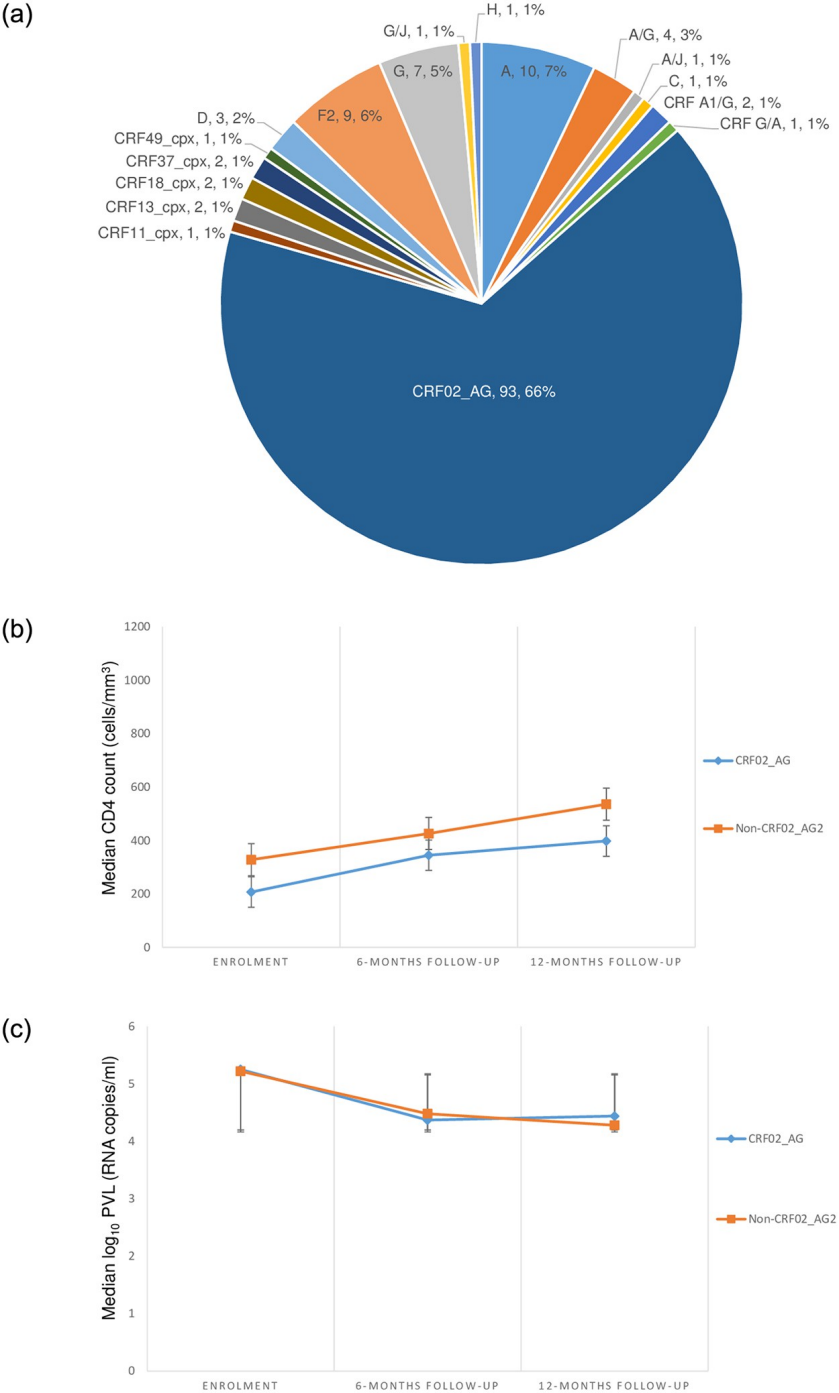
**a:** Rich genetic diversity of HIV-1 subtypes and genetic variants in the study population. **b:** Median CD4 count rise over time across subtypes. Error bars denote standard error. **c:** Median log plasma viral load decreases over time. Error bars denote standard error.

The EDCTP-Ready study parent cohort consisted of three hundred and eleven consenting adolescents living with perinatally acquired HIV on first- and second-line ART at enrolment (Table 1). Of these, 141 participants with PVL ≥ 1000 RNA copies/mL, and whose samples were successfully sequenced were included in the current assessment. From T1-T3 respectively, the proportions of sequences successfully obtained were 45.5% (55/121), 91.4% (96/105), and 88.2% (60/68) (Table 1). Overall, the majority (59.6%) of the study participants were female, with a mean (± standard deviation) age of 15(± 3). The mean age at HIV diagnosis was 6 (± 5) years, and the median [IQR] duration on ART was 51 [46–60] months. At T1, T2, and T3 respectively, adherence-level was 67.3%, 51.0%, and 50.0%; and the majority (89.1%, 77.1%, 66.7) of the participants were on first-line treatment (Table 2). Most of the participants (34.5%, 31.3%, and 31.7%) were on a TDF + 3TC + EFV-based regimen (Table 2). There was an increased rate of switch to second-line protease inhibitor-based ART (10.9%, 22.9%, and 33.3%, p = 0.0007) favored by the presence of acquired drug resistance mutations to previous treatment (Table 2). From T1-T3 respectively, there were declining rates of immunological failure (IF) (49.1%, 32.3%, and 18.3, p < 0.0001), and acquired drug resistance (96.4%, 91.7%, and 85.0%, p = 0.099) (Table 2) (Fig 1a). Acquired drug resistance was driven by the drug classes of non-nucleoside reverse transcriptase inhibitors (96.4%, 88.5%, and 85.0%) (Fig 1b). Likewise, there was a decrease in the proportion of participants with high viremia (52.7%, 28.1%, and 31.7%, p < 0.0001) (Table 2). Seventeen viral clades were identified in this study, with a predominance of the CRF02_AG variant (66.0%) (Fig 2a).

**Table 2:** Demographic, biological, and clinical characteristics of the study population.

	Enrolment (T1)		6-months (T2)		12-months (T3)	
	Frequency	Percentage	Frequency	Percentage	Frequency	Percentage
	n = 55	(%)	n = 96	(%)	n = 60	(%)
**Age (years)**						
10–14	25	45.5	39	40.6	23	38.3
15–19	30	54.5	57	59.4	37	61.7
**Gender**						
Male	25	45.5	40	41.7	21	35.0
Female	30	54.5	56	58.3	39	65.0
**Clinical stage**						
I/II	49	89.1	77	80.2	57	95.0
III/IV	6	10.9	19	19.8	3	5.0
**ART line**						
First	49	89.1	74	77.1	40	66.7
Second	6	10.9	22	22.9	20	33.3
**Adherence**						
Good	37	67.3	49	51.0	30	50.0
Poor	18	32.7	47	49.0	28	46.7
**CD4 classes**						
≥250	18	32.7	64	66.7	45	75.0
<250	27	49.1	31	32.3	11	18.3
**PVL classes**						
≥5 log_10_	29	52.7	27	28.1	19	31.7
<5 log_10_	20	36.4	69	71.9	40	66.7
**Acquired drug resistance**						
Yes	53	96.4	88	91.7	51	85.0
**No**	2	3.6	8	8.3	9	15.0
Median [IQR] CD4 count (cells/mm^3^)	213 [154–313]		366 [309–469]		438 [364–569]	
**Median [IQR] log PVL (RNA copies/mL)**	5.23 [4.71–5.43]		4.43 [4.26–4.64]		4.43 [4.09–4.76]	
**ART regimen**						
**First line RTI-based** *						
ABC + 3TC + EFV	10	18.2	22	22.9	9	15.0
ABC + 3TC + NVP	1	1.8	-	-	-	-
AZT + 3TC + EFV	6	10.9	4	4.2	3	5.0
AZT + 3TC + NVP	10	18.2	17	17.7	9	15.0
D4T + 3TC + NVP	1	1.8	-	-	-	-
TDF + 3TC + EFV	19	34.5	30	31.3	19	31.7
TDF + 3TC + NVP	2	3.6	1	1.0	-	-
**Second line PI/r-based** **						
ABC + 3TC + LPV/r	1	1.8	2	2.1	2	3.3
ABC + 3TC + ATV/r	-	-	1	1.0	1	1.7
AZT + 3TC + LPV/r	-	-	6	6.3	4	6.7
AZT + 3TC + ATV/r	-	-	-	-	1	1.7
TDF + 3TC + ATV/r	3	5.5	11	11.5	8	13.3
TDF + 3TC + LPV/r	1	1.8	2	2.1	4	6.7
TDF + DDI + LPV/r	1	1.8	-	-	-	-

*Reverse transcriptase inhibitor-based antiretroviral therapy.

**Ritonavir boosted protease inhibitor-based second line antiretroviral therapy.

A rich diversity of HIV-1 viral clades was observed, with a mixture of both pure subtypes (A, C, D, F2, G, and H), and recombinant variants (A/G, A/J, CRF A1/G, G/J, CRF G/A, CRF02_AG, CRF11_cpx, CRF13_cpx, CRF18_cpx, CRF37_cpx, and CRF49_cpx) (Fig 2a). For data analysis, these viral clades were grouped into two distinct classes, CRF02_AG and non_CRF02_AG (consisting of subtypes A, C, D, F2, G, H; and variants A/G, A/J, CRF A1/G, G/J, CRF G/A, CRF11_cpx, CRF13_cpx, CRF18_cpx, CRF37_cpx, and CRF49_cpx).

At enrolment, there was no statistically significant difference between the median CD4 count, log_10_ PVL, and ART line across subtypes (p > 0.05). The majority (75.9%) of the participants were on reverse transcriptase inhibitor (RTI) -based first-line regimens (Tables 1 and 2), and the distribution of median log_10_ PVL was similar across subtypes over time (p > 0.05). The median [IQR] CD4 count at enrolment was higher among participants harboring non-CRF02_AG viral clades (328 [112–759] cells/mm^3^), as compared to those with the CRF02_AG variant (207 [33–313]). A similarly variable distribution in median log_10_ PVL across subtypes was observed with a lower value among participants harboring non-CRF02_AG clades (5.22 [4.57–5.72]), relative to those with the CRF02_AG variant (5.25 [4.52–5.49]). However, this variability across subtypes was not statistically significant, p>0.05 (Table 3).

**Table 3:** Socio-demographic and clinical data of study population at enrolment, 6-months, and 12-months follow-up.

		Subtype						
		CRF02_AG		Non-CRF02_AG		Total		p-value
		Count	%	Count	%	Count	%	
**Frequency**		93	66.0%	48	34.0%	141	100%	
**Age ranges (years)**	**10–14**	36	62.1%	22	37.9%	58	100.0%	0.415
	**15–19**	57	68.7%	26	31.3%	83	100.0%	
**Gender**	**Female**	56	67.5%	27	32.5%	83	100.0%	0.650
	**Male**	37	63.8%	21	36.2%	58	100.0%	
**ART line**	**1** ^**st**^	69	64.5%	38	35.5%	107	100.0%	0.513
	**2** ^**nd**^	24	70.6%	10	29.4%	34	100.0%	
**Mean age (±SD)**		15 (3)		15 (3)		15 (3)		
**Median [IQR] duration on ART in months**		49 [44–56]		62 [48–88]		51 [46–60]		
**Median [IQR] CD4 count (cells/mm** ^**3**^ **) at T1**		207 [33–313]		328 [112–759]		213 [75–445]		0.145
**Median [IQR] log**_**10**_ **PVL (RNA copies/mL) at T1**		5.25 [4.52–5.49]		5.22 [4.57–5.72]		5.23 [4.56–5.51]		0.681
**Median [IQR] CD4 count (cells/mm** ^**3**^ **) at T2**		345 [186–600]		426 [195–717]		366 [186–624]		0.240
**Median [IQR] log**_**10**_ **PVL (RNA copies/mL) at T2**		4.37 [3.86–5.13]		4.48 [3.77–5.06]		4.43 [3.82–5.09]		0.947
**Median [IQR] CD4 count (cells/mm** ^**3**^ **) at T3**		398 [286–683]		536 [364–701]		438 [312–700]		0.320
**Median [IQR] log**_**10**_ **PVL (RNA copies/mL) at T3**		4.44 [3.81–5.21]		4.28 [3.62–5.23]		4.43 [3.73–5.21]		0.987

1^st^ line: 1 nucleoside reverse transcriptase inhibitor (NRTI) + 2 non-nucleoside reverse transcriptase inhibitors (NNRTIs)

2^nd^ line: 2 nucleoside reverse transcriptase inhibitors + 1 ritonavir-boosted protease inhibitor (PI/r).

At 6-months post-enrolment, improving values in median CD4 count were observed with a higher value among non-CRF02_AG clades (426 [195–717]), in comparison to the CRF02_AG variant (345 [186–600]). These were accompanied by a declining trend in median log_10_ PVL, with a lower value observed in participants infected with the CRF02_AG variant (4.37 [3.86–5.13]), relative to those with non-CRF02_AG clades (4.48 [3.77–5.06]). Likewise, this difference was not statistically significant with respect to genetic variability (Table 3). The proportion of participants who had an adequate immunological response, as well as those with PVL < 5 log was comparable across subtypes with p-value > 0.05 (Tables 4 and 5).

**Table 4:** Immunological response at 6-, and 12-months post-enrolment with respect to viral genotypes.

		Subtype							
		CRF02_AG		Non-CRF02_AG		Total		OR(95%CI)	p-value
		Count	%	Count	%	Count	%		
**CD4 cell count at Enrolment (cells/mm** ^**3**^ **)**	**<250**	20	74.1%	7	25.9%	27	100.0%	2.86(0.81–10.10)	0.122
	**≥250** *	9	50.0%	9	50.0%	18	100.0%		
**CD4 cell count at 6-months (cells/mm** ^**3**^ **)**	**<250**	23	74.2%	8	25.8%	31	100.0%	1.40(0.54–3.66)	0.636
	**≥250**	43	67.2%	21	32.8%	64	100.0%		
**CD4 cell count at 12-months (cells/mm** ^**3**^ **)**	**<250**	8	72.7%	3	27.3%	11	100.0%	1.47(0.34–6.34)	0.732
	**≥250**	29	64.4%	16	35.6%	45	100.0%		

*Favorable immunological response: CD4 count ≥ 250 cells/mm^3^.

**Table 5:** Virological response with respect to genetic diversity at enrolment, months 6 and 12.

		Subtype							
		CRF02_AG		Non-CRF02_AG		Total		OR	p-value
		Count	%	Count	%	Count	%		
**PVL at Enrolment (RNA copies/mL)**	>5 log	17	58.6%	12	41.4%	29	100.0%	0.76(0.24–2.48)	0.769
	<5 log	13	65.0%	7	35.0%	20	100.0%		
**PVL at 6-months (RNA copies/mL)**	>5 log	19	70.4%	8	29.6%	27	100.0%	1.04(0.39–2.75)	1.000
	<5 log	48	69.6%	21	30.4%	69	100.0%		
**PVL at 12-months (RNA copies/mL)**	>5 log	12	63.2%	7	36.8%	19	100.0%	0.83(0.26–2.59)	0.775
	<5 log	27	67.5%	13	32.5%	40	100.0%		

At 12-month follow-up, there was a further increase in median CD4 count with a better outcome observed among non-CRF02_AG-infected participants (536 [364–701]), as opposed to those with the CRF02_AG variant (398 [286–683]). Similarly, there was a declining trend in median log_10_ PVL, with a lower value observed in non-CRF02_AG clades (4.28 [3.62–5.23]), as compared to that in the CRF02_AG variant (4.44 [3.81–5.21]). This difference was not statistically significant with respect to HIV-1 subtypes/genetic variants (Table 3).

Concerning median CD4 count, all subtypes showed a consecutive rise over time (Fig 2b). Likewise, a steady decrease in median PVL was observed from enrolment to the end of the observation period (Fig 2c). At the end of the study, no statistically significant differences with respect to subtype were observed in both participants who experienced adequate immunological response (CD4 count ≥ 250 cells/mm^3^), and those with PVL< 5 log_10_ (Tables 4 and 5).

By the end of the assessment period, 22.2% (28/126) of the participants experienced immunological failure (CD4 < 250 cells/mm^3^), and 34.3% (47/137) had PVL > 5 log_10_ RNA copies/mL (Tables 4 and 5). Concerning progression to study endpoints, 18.3% vs. 4.1% of participants infected with the CRF02_AG variant and non-CRF02_AG clades respectively had CD4 cell count < 250 cells/mm^3^ at the end of follow-up. Likewise, proportions of 22.6% and 11.7% of participants harboring CRF02_AG and non-CRF02_AG infections respectively had PVL > 5 log_10_ RNA copies/mL (Table 6).

**Table 6:** Proportion of participants progressing to each outcome by HIV-1 subtype.

Subtype(s)/genetic variant(s)	Outcome (%)							
	CD4 cell count <250 cells/mm^3^				Plasma viral load > 5 log RNA copies/mL			
	Enrolment	6-months	12-months	Entire follow-up	Enrolment	6-months	12-months	End of follow-up
**CRF02_AG**	20 (44.4)	23 (24.2)	8 (14.3)	23 (18.3)	17 (34.7)	19 (19.8)	12 (20.3)	31 (22.6)
**Non-CRFO2_AG**	7 (15.6)	8(8.4)	3 (5.4)	5(4.1)	12 (24.5)	8 (8.3)	7 (11.9)	16 (11.7)

**Fig 3:**
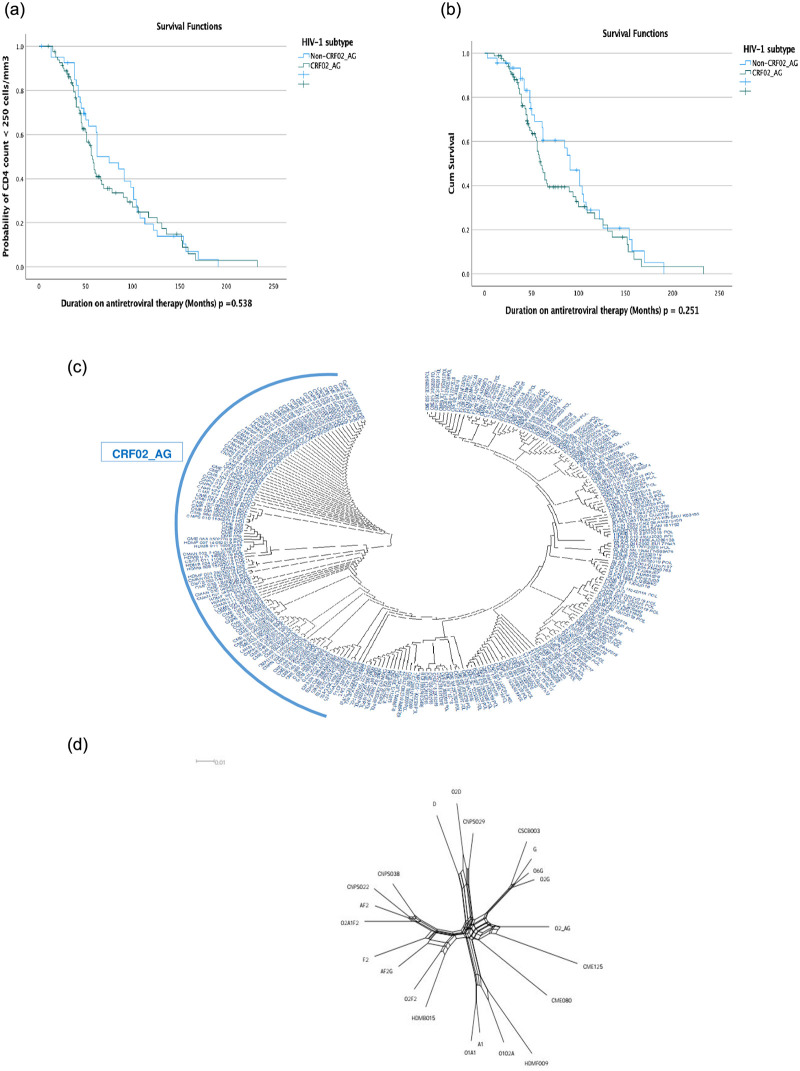
**a:** Kaplan Meier analysis of time estimate to reach CD4 count < 250 cells/mm^3^. **b:** Probability of plasma viral load > 5 log RNA copies/mL over time. **c:** Maximum likelihood phylogenetic tree of the Protease-Reverse transcriptase gene regions of study participant sequences. This depicts broad genetic diversity with a wide inter/intra subtype recombination. **d:** Splits Tree analysis results: Maximum likelihood phylogenetic tree of the Protease-Reverse transcriptase gene regions of the four (CNPS029, CME125, HDMF009, and HDMB015) inter-subtype recombinants identified in the study.

The overall median time to poorer immunologic outcome (CD4 < 250cells/mm^3^) was 61.00 (95% confidence interval, CI: 55.83–66.17) months. Participants infected with the CRF02_AG variant (57.00: 95% CI: 51.67–62.33 months) had a shorter median time to a poor immunologic outcome as compared to those infected by non-CRF02_AG clades (62.00: 95% CI: 38.88–85.12 months). However, these differences in the survival curves of the different subtypes/variants were not statistically significant, p = 0.538 (Fig 3a).

Concerning virological response, the median time for the participants to experience high viral load (PVL > 5 log_10_ RNA copies/mL) was 64.00 (95% CI: 41.74–86.26) months. Those with CRF02_AG infection had a shorter median time to this endpoint (60.00: 95% CI: 52.72–67.28 months) as compared to participants infected by non-CRF02_AG clades (91.00: 95% CI: 75.38–106.62 months). The observed differences in survival curves across subtypes were not statistically significant, with p = 0.251 (Fig 3b).

Over the entire study period, the duration of ART was significantly associated with a decrease in the probability of experiencing poorer immunologic outcomes among participants infected by the CRF02_AG variant at any given time over the two-year duration of the study [adjusted hazard ratio (aHR) = 0.02, 95% confidence interval (95% CI): 0.001–0.52, and p = 0.017]. Similarly, there was a statistically significant association between the duration of treatment and the probability of a decline in PVL in participants with CRF02_AG infection [aHR = 0.05, 95% CI: 0.01–0.47, and p = 0.008] (Table 7).

**Table 7:** Cox regression modelling outcome: CD4 < 250 cells/mm3, and PVL > 5log10 RNA copies/ml.

		CD4 cell count <250 cells/mm^3^				Plasma viral load >5 log_10_ RNA copies/mL			
		Unadjusted analysis		Adjusted analysis for other variables		Unadjusted analysis		Adjusted analysis for other variables	
		HR (95% CI)	p-value	HR (95% CI)	p-value	HR (95% CI)	p-value	HR (95% CI)	p-value
**Non-CRF02_AG**									
	**Acquired drug resistance**	24.82 (0.00–7482181.61)	0.618	90.58 (0.00–2.34E+34)	0.906	23.66 (0.01–49085.37)	0.417	1787.95 (0.00–1.86E+65)	0.918
	**Gender**	1.74 (0.24–12.45)	0.579	0.20 (0.00–84.83)	0.603	5.11 (1.59–16.38)	0.006	42.53 (0.07–27756.68)	0.257
	**ART line**	1.11 (0.12–9.99)	0.923	0.07 (0.00–170.04)	0.502	1.83 (0.64–5.28)	0.263	0.63 (0.04–9.31)	0.734
	**Adherence**	0.42 (0.07–2.62)	0.356	9.11 (0.00–47897.09)	0.613	1.13 (0.41–3.23)	0.821	9.65 (0.03–3565.85)	0.452
	**Duration on treatment**	0.85 (0.74–0.99)	0.035	0.34 (0.08–1.42)	0.139	0.86 (0.79–0.93)	<0.001	0.11 (0.01–2.05)	0.134
**CRF02_AG**									
	**Acquired drug resistance**	0.23 (0.08–0.71)	0.009	0.03 (0.00–446.84)	0.464	0.31 (0.10–0.88)	0.028	0.46 (0.01–34.25)	0.722
	**Gender**	1.29 (0.55–2.99)	0.558	1.34 (0.21–8.60)	0.761	1.66 (0.81–3.38)	0.165	0.78 (0.24–2.48)	0.668
	**ART line**	1.42 (0.59–3.38)	0.435	3.05 (0.47–19.88)	0.243	1.01 (0.45–2.26)	0.988	0.64 (0.18–2.31)	0.495
	**Adherence**	1.00 (0.42–2.37)	0.996	2.67 (0.34–21.17)	0.353	1.47 (0.68–3.20)	0.333	1.74 (0.51–6.04)	0.385
	**Duration on treatment**	0.85 (0.79–0.92)	<0.001	0.02 (0.001–0.52)	0.017	0.84 (0.79–0.91)	<0.001	0.05 (0.01–0.47)	0.008

Assessing potentially emerging HIV-1 genetic variants, or unique recombinant forms (URF) associated with reduced viral susceptibility to currently available ARV drugs in the Cameroonian context, using four rapid subtyping tools revealed a high rate of inter-subtype recombination, confirmed by phylogenetic analyses (Fig 3c), with the identification of eight discordant potential unassigned URFs. From these, further analyses to detect and analyze recombination and/or genomic assortment signals using RDP4 revealed four potential URFs with distinct breakpoints, each associated with major drug resistance mutations mainly driven by the ARV drug classes of non-nucleoside reverse transcriptase inhibitors (NNRTIs) and nucleoside reverse transcriptase inhibitors (NRTIs) (Table 8). Further phylogenetic analysis suggest the presence of inter-subtype recombinants; recombinant CRF02_AG/02D, recombinant CRF02_AG/02A1F2, recombinant D/CRF02_AG, and recombinant AF2/CRF02_AG (Fig 3d).

**Table 8:** Potential HIV-1 Unique recombinant forms (URFs) with unique breakpoints and associated major drug resistance mutations according to ARV drug classes.

Seq. ID	Subtypes/genetic variants			Major drug resistance mutations (DRMs)		
	Major Parent	Minor Parent	Conclusion	Major NRTI	Major NNRTI	Major PI/r
CME_125 (OR259825)*	CRF02_AG	02D	Recombinant CRF02_AG/02D	K70KR	K103N, Y181C, F227L	None
HDMB_015 (OR259967)	CRF02_AG	02A1F2	Recombinant CRF02_AG/02A1F2	M184V	K103N, V108VI	None
CNPS_029 (OR259851)	D	CRF02_AG	Recombinant D/CRF02_AG	M184V	K103N, Y188YC, P225H	None
HDMF_009 (OR259781)	AF2	CRF02_AG	Recombinant AF2/CRF02_AG	D67N, K70R, M184V, T215V, K219Q	V108I, Y181C, G190A, F227FL	None

• Accession number in GenBank

## 4. Discussion

Our study was conducted among adolescents living with perinatally acquired HIV (APHI) in urban and rural settings of the Center region of Cameroon. The predominant genetic variant was CRF02_AG, this is similar to other studies carried out in Cameroon [4, 6, 9, 19–21]. The CRF02_AG variant is the most prevalent HIV-1 viral clade in West Africa [1]. It is a recombinant of pure subtypes A and G, which are both associated with slower disease progression to AIDS [5]. The higher infectivity observed in infection with this variant may be attributed to its enhanced replicative fitness over its pure subtype constituents and hence improved adaptation to its host. HIV-1 subtypes C, D, and the genetic variant CRF01_AE observed in our study have been associated with faster HIV-1 disease progression to AIDS [7, 10, 22, 23] and advanced immunodeficiency. Evidence suggests that recombinant forms have a faster rate of disease progression when compared to pure lineages. This is because they have better replicative fitness as compared to their parental forms [5]. However, the immune-virological response in this evaluation was similar across viral clades, despite the presence of a wide array of circulating recombinant forms and unique recombinant forms (CRF18_cpx, CRF49_cpx, G/J, CRF A1/G, CRF G/A, CRF11_cpx, CRF13_cpx, CRF37_cpx).

In this study, there was no statistically significant difference across subtypes with respect to CD4 cell count at enrolment. Likewise, there was no significant difference in the proportion of participants who achieved adequate immunological response at 12 months post-enrolment. These results were similar to those obtained in other studies [24, 25]. Moreover, assessing endpoint events in our study revealed that duration on ART was significantly associated with the rate of progression to poorer immunological outcomes among participants with CRF02_AG infection. It should be noted that the focus of the majority of studies of inference on the effect of subtype on immunologic decline has been focused on patients’ pre-exposure to ART at baseline. Additionally, most focus on comparing patients harboring subtype B versus non-B subtypes, hence grouping minority variants into non-B subtypes. This makes it difficult to assess the effect of these less predominant viral clades. Studies carried out in China, where variant CRF01_AE is the most predominant clade, revealed that this variant is associated with fast progression to AIDS (with a shorter median time from the estimated date of seroconversion to AIDS) and advanced immunodeficiency (shorter median time from estimated date of seroconversion to CD4 cell count <100 cells/μl) as compared to non-CRF01_AE viral clades [22, 23]. However, a study in Nigeria reported similar rates of CD4 cell count recovery across all subtypes [24], which is concordant with the results obtained in our evaluation. In contrast, a previous study revealed that patients infected with either a CRF02_AG strain or another non-B viral clade had better immunological responses than those infected with a subtype-B virus [26]. Despite the absence of statistical significance in the rate of progression to poorer immunologic outcome in this evaluation, HIV-1 genotypes such as subtypes C and D, and variant CRF01_AE were identified in our study population. These have previously been associated with faster disease progression [22, 23, 27], likely due to faster rates of CD4 T-cell decline. Additionally, a higher probability of having a virus with a CXCR4 tropism in subtype D infections has been reported. Conversely, subtypes A and G have been associated with a less aggressive disease progression [7]. Further analysis into the rise in median CD4 cell count over time, as well as the estimated time to achieve favorable immunologic response in our study, showed similar responses across all subtypes. These results agree with those of a previous study carried out in a West African country [24]. However, they are discordant with results obtained in geographical regions outside West-Central Africa [26–29].

Virological responses at enrolment, 6-, and 12-month follow-ups on treatment were comparable across all subtypes. These were similar to results obtained by Ogbenna *et al* [24] but in disagreement with those obtained by Chaix *et al* in France where infection with CRF02_AG and other non-B viral clades conferred better virological responses than patients harboring B-subtypes [26]. These findings of similar virological responses across subtypes are consistent with those observed in previous studies that compared the responses to ART stratified by HIV-1 subtype [24, 26, 28, 30]. Our study also showed a continuous decrease in median PVL over time, as well as comparable overall virologic responses across subtypes. Similar observations have been reported in other studies [24, 26, 28, 29].

In our study, we identified four sequences with discordant subtypes according to the rapid subtyping tools used, which we classified as potential URFs. In addition, each of these sequences was associated with at least one major drug-resistant mutation. These results are in agreement with those observed in several other studies carried out in Cameroon [4, 9, 19, 31, 32]. This study provided insight into the evolving HIV-1 diversity, the genomic complexity of URF infections, the structural dynamics of emerging recombinant forms, and possible implications for antiretroviral treatment. Thus, underlining the necessity of performing full/near-full length next-generation sequencing to achieve accurate HIV surveillance, subtyping, and genotypic analyses.

As generally observed in longitudinal studies, there were some missing laboratory data over time, as well as loss to follow-up of participants by the end of the study which made it difficult to follow-up the entire sample population. These may have had an impact on data quality and the overall impact of the study findings on the target population. Furthermore, limiting sequencing only to samples that had PVL ≥ 1000 RNA copies/ml and sequencing failure of some samples led to an underestimation of HIV genotypes. Additionally, further analyses like Simplot++ ought to be performed to investigate sequence similarity and confirm the detection of recombination events. However, the multi-time point follow-up period, the personalized monitoring, and repeated genotyping of participants to ensure within-program comparison of subtypes in a bid to limit expected confounders are the strengths of this study.

## 5. Conclusion

This study revealed a wide variety of HIV-1 genotypes with CRF02_AG predominance. Among participants with CRF02_AG infection, duration on antiretroviral treatment was significantly associated to both rates of progression to poorer immunological outcomes, and higher PVL following treatment failure. Virological outcomes were similar across HIV-1 subtypes/variants. Therefore, provided adequate observance of treatment, and personalized follow-up of this underserved adolescent population with particular importance given to immunological follow-up, current ART regimens have similar efficacy irrespective of subtype.
